# Effect of training about child neglect and abuse to teachers and its effect to awareness

**DOI:** 10.1186/s12889-022-12904-8

**Published:** 2022-03-19

**Authors:** İskender Gün, Adeviye Çopur, Elçin Balcı

**Affiliations:** 1 Erciyes University Faculty of Medicine, Public Health Department, 38039 Kayseri, Turkey; 2 Kayseri University, Incesu Vocational High School, Kayseri, Turkey

**Keywords:** Child neglect, Child abuse, Schoolteachers, High schools, Education, Turkey

## Abstract

**Background:**

This study aimed to evaluate the effects of child neglect and abuse training on the knowledge and awareness of medical vocational schoolteachers in Turkey.

**Methods:**

This study was conducted based on data taken from a sample of teachers from Kayseri, Turkey, between October 2016 and April 2017. Teachers who agreed to participate in the study received training pertaining to child neglect and abuse. Data were collected through a survey form and the Scale for Identifying the Symptoms and Risks of Child Abuse and Neglect. Data were analyzed using SPSS V.20.0 software. For statistical analyses, Student’s t test, analysis of variance and McNemar tests were conducted, with a value of *p* <  0.05 being considered statistically significant.

**Results:**

Of teachers in the sample, 63.7% were female, and 80.5% were married. Teachers’ mean age was 40.5 ± 9.9 years. Among teachers, 87.4% reported that the training was sufficient. Teachers’ level of knowledge concerning neglect and abuse and the percentage of teachers who reported that they would report such situations to the authorities when faced with such a case increased after training. Female teachers’ scores on the neglect and abuse scale increased among teachers without children and those who found the training to be sufficient.

**Conclusions:**

The knowledge level of teachers in the study group was found to be increased compared to their knowledge level prior to training. The subject of child neglect and abuse should be further discussed in the context of teachers’ formal training.

## Background

Child neglect and abuse is a global issue. Neglect and abuse can cause physical, mental, sexual and social harm to the child and endanger his or her health and safety [1, 2]. Child abuse can be classified as the physical, emotional, psychological or sexual abuse of children or as the neglect of children [3].

Physical abuse is defined as the intentional use of physical force against a child that results in harm to the child’s health, development, or dignity [3]. Emotional or psychological abuse involves both isolated incidents and a pattern of failure over time on the part of a parent or caregiver to provide a developmentally appropriate and supportive environment [3]. These incidents are typically repeated and convey to the child that the child is worthless, unloved, unwanted or only valuable in terms of meeting another’s needs, and they are exemplified by acts of hostility, terrorizing, rejection, isolation, corruption and denying emotional responsiveness [4]. Sexual abuse is defined as the involvement of a child in sexual activity by any adult or child in a position of power over the victim [3, 5]. Neglect includes a pattern of failure over time on the part of a parent or other family member to provide for the development and well-being of the child in terms of health, education, emotional development, nutrition, shelter, and safe living conditions [3, 6].

Specifically, child abuse has become a grave and increasing problem worldwide. According to data released by UNICEF, 42.0% of students in Australia stated that they had been subjected to child abuse [7]. In Poland, 80.0% of adults declared themselves to have been victims of physical violence in their childhood [8].

In Turkey, the main factors resulting in an increase in the number of children exposed to neglect and abuse are described as follows: the vast number of families with low socioeconomic status, a high population growth rate, the fact that individuals use violence as a form of discipline and increasing levels and rates of unemployment and immigration [9]. Furthermore, a study conducted in Turkey demonstrated that child abuse mostly occurs within the child’s home, school or in the street and that abusers are mostly fathers, mothers, teachers, friends and neighbors [10]. When children were asked to identify what type of abuse they had witnessed, 56.0% reported witnessing physical abuse, 49.0% reported witnessing emotional abuse and 10.0% reported witnessing sexual abuse; however, these numbers changed when they were asked about what type of abuse they had experienced, as 25.0% reported experiencing neglect, 51.0% reported experiencing emotional abuse and 3% reported experiencing sexual abuse [10].

Generally, child neglect and abuse may result in various injuries to the child or even death; in addition, children who carry on with their lives after an incident of abuse may experience several mental problems, and drug addiction or an inclination toward crime and prostitution may be observed in these cases [7]. Furthermore, children in early childhood who are exposed to neglect and abuse are more likely to abuse other children in their close environment when they become adults, thereby contributing to a vicious cycle (as this behavior is repeated by subsequent generations): a previous study has confirmed this claim, as mothers and fathers who experienced abuse early in their lives also abuse their own children, and the study concludes that what truly matters is children being safe from neglect, abuse and other types of harm [7].

To keep children safe, it is necessary to identify symptoms of child neglect and abuse and to ensure that legal notices regarding this type of behavior are filed in accordance with legal procedures. Like other civil servants, teachers have certain responsibilities regarding child abuse, and they are obliged to file legal notice in case they witness such incidents [11, 12]. Furthermore, studies have shown that teachers play a crucial role in recognizing and preventing child abuse because they are often the first professionals to have a long-term close relationship with children, and they communicate with children in this setting [1, 13]. Thus, alongside preventive efforts, teachers should also take on the task of identifying children who are victims of abuse and reporting these cases to the relevant authorities [13, 14].

According to Turkish penal code article 280, all civil servants must file legal notice if they learn about child abuse cases as soon as possible. Similarly, health professionals have particular liabilities with respect to filing legal notice in such cases [15]; since health professionals participate in health institutions where there is a possibility of identifying children neglect and abuse, they are expected to be competent with respect to such identification [2]. Accordingly, since teachers at medical vocational schools raise and teach those who may become future health personnel, it is of significant importance that they are knowledgeable and qualified on the topic.

## Methods

### Aim

This study aimed to determine the effects of child neglect and abuse training on the knowledge and awareness of Turkish medical vocational schoolteachers.

### Materials

This study was a descriptive study focusing on teachers working at medical vocational schools in Kayseri, Turkey. In Turkey, medical vocational schools produce assistant nurses. The study was carried out between October 2016 and April 2017. During the period of this study, 305 medical vocational teachers worked in 12 schools (four private and eight public schools) in the city of Kayseri, and all of these teachers were invited to participate. During data collection, ten teachers were on leave, and 80 teachers did not agree to participate in the study; these cases were either excluded from the study or did not participate at all, so the final sample comprised 215 teachers from 12 schools.

An unpublished study was conducted by the researcher between April and September 2016 to identify the levels of knowledge concerning child neglect and abuse among teachers working in Turkish private and public medical vocational high schools. Teachers who participated in the previously conducted study stated that they did not have sufficient knowledge to identify neglect and abuse and, if possible, that they would like to participate in trainings related to this topic.

Thus, the researcher developed child neglect and abuse training for the same group. To increase their level of competence with respect to this issue, individual training pertaining to child neglect, types of abuse and the legal framework was provided by the Forensic Medicine Department of one of the researchers. Training was administered in the teachers’ own school schools. The educational content included the symptoms of child abuse and neglect, the legal dimensions of this situation and what the teacher can do when faced with such a situation. Education was provided through a group approach and took place over approximately 30 min. After the training, a questionnaire and scale were administered via a face-to-face interview technique.

For data collection, a questionnaire containing 15 questions pertaining to the socioeconomic characteristics of teachers, a self-evaluation of the adequacy of the training provided and the Scale Form for Identifying the Symptoms and Risks of Child Abuse and Neglect developed by Uysal [16] were employed. The scale was used with the permission of its owner.

Moreover, a validity-reliability study was conducted for the aforementioned scale, which is a Likert-type scale consisting of 67 items. In this scale, there are six subgroups: physical symptoms of abuse on the child (19 items), behavioral signs of child abuse from the child (15 items), signs of neglect from the child (7 items), neglected and abusive parent characteristics (13 items), characteristics of children susceptible to neglect and abuse (5 items), and familial features related to child neglect and abuse (8 items). The scale’s internal consistency was calculated, and the Cronbach’s alpha value was 0.924. Questions numbered 3, 5, 8, 10, 12, 14, 16, 27, 28, 30, 32, 34, 41, 42, 46, 49, 52, 54, 56, 59, 61, 63 were negatively oriented questions. Scores closer to 5 indicated more correct answers, and scores farther away from 5 indicated more incorrect answers. Participants who provided correct answers to all items received a full score of 335.

The data collected were evaluated using SPSS V 20.0 software. For statistical analyses, Student’s t test, analysis of variance (ANOVA), the chi square test and the McNemar test were used, and results with a value of *P* <  0.05 were considered to be statistically significant.

## Results

Teachers’ mean age was 40.5 ± 9.9 years, and their mean work experience was 15.7 ± 11.0 years. Of the teachers in the study, 87.4% stated that the researcher’s training was sufficient; there was no significant difference regarding this topic between teachers at public schools and those at private schools. The rate of teachers who were married and had children was higher for teachers in public schools. In addition, the service period of teachers working in the public sector was longer. While the gender ratios for teachers in public schools were fairly even, the proportion of women in private schools was higher. While the rate of teachers from health backgrounds was higher in public schools, the rate of teachers from other fields in private schools was found to be higher. The differences among all these variables were found to be statistically significant (Table 1).

**Table 1:** Various sociodemographic characteristics of teachers according to their schools

		School				Total		Chi square	p
		Private		Public					
		Number	%	Number	%	Number	%		
Marital status	Married	39	55.7	134	92.4	173	80.5	40.451	< 0.001
	Single	31	44.3	11	7.6	42	19.5		
Gender	Male	15	21.4	63	43.4	78	36.3	9.902	0.002
	Female	55	78.6	82	56.6	137	63.7		
Has a child	Yes	34	48.6	131	90.3	165	76.7	46.158	< 0.001
	No	36	51.4	14	9.7	50	23.3		
Occupational branch of teaching	Health	29	41.4	82	56.6	111	51.6	4.323	0.042
	Other	41	58.6	63	43.4	104	48.4		
Work duration (years)	0–10	51	72.9	28	19.3	79	36.7	68.921	0.001
	11–20	6	8.6	33	22.8	39	18.1		
	21–30	5	7.1	75	51.7	80	37.2		
	31 and over	8	11.4	9	6.2	17	7.9		
	Total	70	100.0	145	100.0	215	100.0		

The ratio of teachers who thought they had enough knowledge to recognize child neglect and abuse after the training increased from 40.9 to 82.3%, and this change was found to be statistically significant (Table 2).

**Table 2:** Teachers’ self-reported levels of adequate knowledge with respect to identifying child neglect and abuse before and after training

View concerning whether they had adequate knowledge to identify child neglect and abuse				
	Before Training		After Training	
	Number	%	Number	%
Yes	88	40.9	177	82.3
No	127	59.1	38	17.7
Total	215	100.0	215	100.0

McNemar *P* < 0.001

The ratio of teachers who believed they would file legal notice when faced with child neglect and abuse after the training increased from 14.4 to 22.8%, and this change was found to be statistically significant (Table 3). The main reason for not filing a legal notice was stated to be a lack of knowledge among teachers before training.

**Table 3:** Teachers’ views regarding whether they would file legal notice when faced with child neglect and abuse before and after training

Teachers’ views concerning whether they would file legal notice when faced with child neglect and abuse				
	Before Training		After Training	
	Number	%	Number	%
Those who stated they would give a legal notice	31	14.4	49	22.8
Those who stated they would not do so	184	85.6	166	77.2
Total	215	100.0	215	100.0

McNemar *P* = 0.033

Then, we evaluated teachers’ total scores according to various variables, which showed that female teachers’ total scores (258.3) were higher than those of male teachers (247.6), and the difference between genders was statistically significant. The total score of teachers who believed that “the training was sufficient” was high (255.9 versus 232.3), and this difference was statistically significant. Furthermore, we evaluated teachers’ total scores according to their work experience, age, and whether they had children or reported abuse, and there were no statistically significant differences for these factors. Teachers who had a child, would file a legal notice and were under 35 years of age had higher scores, but this difference was not statistically significant (Table 4).

**Table 4:** Teachers’ overall total scores on the Scale Form for Identifying the Symptoms and Risks of Child Neglect and Abuse according to demographic characteristics

		Overall Total Score		t	P
		Number	Mean ± SD		
Gender	Male	78	247.6 ± 23.7	−3.109	0.002
	Female	137	258.3 ± 24.6		
Has a child	Yes	165	253.7 ± 25.2	−0.736	0.304
	No	50	256.7 ± 23.1		
Would file legal notice	Yes	49	259.7 ± 21.7	1.856	0.067
	No	166	252.9 ± 25.4		
View of training	Adequate	188	255.9 ± 24.8	2.491	0.014
	Inadequate	7	232.3 ± 18.8		
Work experience (years)	0–10	79	258.4 24.3	1.181	0.318
	11–20	39	251.2 23.6		
	21–30	80	252.9 25.5		
	31 and above	17	250.2 25.3		
Age	35 and below	72	258.0 22.8	1.510	0.132
	36 and above	143	252.6 25.6		

## Discussion

Focusing on teachers working at medical vocational schools in the city of Kayseri in Turkey, this study aimed to identify the effects of child neglect and abuse training on teachers’ knowledge and awareness of child neglect and abuse.

In Turkey, it has been reported that teachers receive limited training pertaining to child neglect and abuse during their formal training [17]. However, a study conducted by Sagir and Gozler showed that Turkish teachers had a moderate level of knowledge concerning this topic, even if 89% of participants reported not receiving any training on the subject [18]. Supported by these studies, our results showed that teachers’ knowledge of how to recognize child neglect and abuse was insufficient: While 40.9% of participants stated that they had sufficient knowledge to identify abuse before training, this rate increased to 82.3% after training. Thus, it was observed that teachers’ awareness regarding child neglect and abuse increased after training. We identified this change as the result of training.

Furthermore, our results showed that before the training, the rate of teachers who reported they would file legal notice when faced with a case of child neglect and abuse was very low (14.4%); this rate increased to 22.8% after training. In the study by Sagir and Gozler [18], 84.4% of teachers thought that they should report cases of child neglect and abuse; in the study by Kurklu [19], this rate was 85.9%. Kurklu’s study showed that a lack of knowledge of legal processes was the reason why teachers did not file legal notice when faced with such a situation [19]. Similarly, our results showed that teachers’ reason for not filing legal notice may be a lack of knowledge concerning the legal aspects of the issue. In contrast, in the study by Ozgul [20], almost all teachers and school administrators stated that they would file legal notice in this context. Uslu and Zincir [2] performed a study including different occupational groups, including teachers. Their findings also support this conclusion, and further examination of the reasons for not filing legal notice showed that most teachers and school administrators were fearful of putting the child in a worse situation than their current situation. Moreover, when the same reasons were examined in the study by Tugay [21], the results showed that these reasons were similar to those found in the study by Uslu and Zincir: teachers were concerned for the child and feared that the child could come to suffer even more after the legal notice because the child abuse would continue. Nevertheless, concerning this topic, Kenny’s study analyzed and compared trained and untrained teachers with respect to whether they would file legal notice when faced with child neglect and abuse [22], and the results showed that teachers who had received training before they started their teaching service filed legal notice more often. Thus, in our study, since the ratios for teachers who would file legal notice indeed increased after training, it is possible to say that these aforementioned studies corroborate our results, mainly because they show that teachers’ most common deficiencies with respect to this specific topic may be due to the inadequacy of training during their university education and their lack of knowledge concerning legal obligations and relevant legal procedures (the two topics directly explored in our training).

When teachers’ total scores on the Scale Form for Identifying the Symptoms and Risks of Child Abuse and Neglect were examined, the results showed that teachers had a good level of knowledge concerning the topic after training. In studies of health care workers, the scores of participants who received training increased after training [23, 24]. Moreover, in a study conducted by Uysal and Ozsoy [25], the mean scores of teachers who received in-service training were significantly higher. Moreover, our results, which showed that teachers evaluated the training as ‘sufficient’, also support the conclusion that better-trained teachers are more able to identify the symptoms of child abuse and neglect.

Our analyses also showed that the total scores of female teachers were higher than those of male teachers. Demir [26], in her study of physicians, reported that female physicians had higher levels of knowledge regarding child neglect and abuse. Even studies performed with respect to different occupational groups agree that this difference can be explained by the fact that women may be more aware of children’s concerns and behaviors as they spend more time with them.

Moreover, when participants’ total scores were analyzed with respect to whether they had children, the total score averages and overall total scores of participants without children were higher. Some researchers performed studies using the Scale Form for Identifying the Symptoms and Risks of Child Abuse and Neglect: Demir [26] used physicians as a study group, while Kocaer [27] employed nurses and physicians, Kara [28] referenced physicians and Yılmaz [29] examined nurses. As another example of corroboration, those studies have shown that individuals who do not have children score higher on the scale [26–29]. Thus, people who have children, even when they work in different professions, may be more inclined to view certain abusive and/or negligent behaviors as normal behaviors in the disciplining and education of children.

In one study, it was demonstrated that the rate of nurses who correctly identify the situations in which they should report neglect and abuse varied between 87 and 97% [30]. In our study, the frequency of identifying situations that should be reported was found to be lower, although this measure was based only on participant self-evaluations. Possible reasons for this large difference may include differences among countries as well as the fact that teachers receive education pertaining to neglect and abuse at a lower rate than nurses. Similar to our study, it was demonstrated by another study that education among health care and education providers increased awareness of abuse and neglect [31].

Study limitations: There was no control group for this study because all the teachers in the sample were included in the study group. Teachers who did not agree to participate in the study may have had different results.

## Conclusion

In sum, teachers’ knowledge levels concerning child neglect and abuse significantly increased after training. Accordingly, an intervention program should be developed and applied within the Turkish national education system to help teachers identify incidents of child neglect and abuse. Furthermore, there is a special need for the development and application of such a scheme in the context of Turkish schools that train health personnel so that neglect and abuse can be avoided in these settings to ensure that future health workers have the tact and the knowledge to recognize and act in such situations, which is an important point mainly due to their level of responsibility as one of the first close contacts between children and adults outside their families in Turkey - and in many countries worldwide.

## Data Availability

The datasets used and/or analyzed during the current study are available from the corresponding author on reasonable request.
